# Diagnostic Value and Outcomes of Systematic SARS-CoV-2 Screening in Asymptomatic Patients

**DOI:** 10.1001/jamanetworkopen.2026.5867

**Published:** 2026-04-08

**Authors:** Marvin Weiss, Pascal Urwyler, Matthias von Rotz, Richard Kuehl, Sabine Kuster, Isabelle Vock, Lisandra Aguilar-Bultet, Fabian C. Franzeck, Claudia Bagutti, Katharina Rentsch, Stefano Bassetti, Karoline Leuzinger, Sarah Tschudin-Sutter

**Affiliations:** 1 Division of Infectious Diseases, University Hospital Basel & University of Basel, Basel, Switzerland; 2 Department of Infectious Diseases, University Medical Center Basel-Land, Liestal, Switzerland; 3 Research and Analytics, Department of Informatics, University Hospital Basel & University of Basel, Basel, Switzerland; 4 State Laboratory, Basel-City, Basel, Switzerland; 5 Laboratory Medicine, University Hospital Basel, Basel, Switzerland; 6 Division of Internal Medicine, University Hospital Basel, Basel, Switzerland; 7 Clinical Virology, University Hospital Basel & University of Basel, Basel, Switzerland; 8 Department of Clinical Research, University Hospital Basel & University of Basel, Basel, Switzerland

## Abstract

**Importance:**

Early detection of SARS-CoV-2 may mitigate nosocomial spread, yet universal screening of asymptomatic patients remains debated.

**Objective:**

To evaluate the diagnostic yield of systematic SARS-CoV-2 screening in correlation with community incidence and wastewater viral load and assess clinical outcomes among inpatients with false-positive results.

**Design, Setting, and Participants:**

This retrospective quality-control cohort study assessed the results of systematic SARS-CoV-2 screening of asymptomatic patients at the University Hospital Basel in Basel, Switzerland, a tertiary care center admitting more than 40 000 adult patients annually. All patients with systematic screenings for SARS-CoV-2 infection from February 8, 2021, to July 5, 2021, and from August 25, 2021, to December 5, 2022, were included. Data were analyzed from January 2024 to February 2025.

**Exposure:**

Saliva-based PCR-tests to screen for asymptomatic SARS-CoV-2 infection on admission and at regular 3- to 5-day intervals during hospitalization.

**Main Outcomes and Measures:**

The primary end points were the proportion of positive and false-positive SARS-CoV-2 test results, as well as the number needed to screen to find 1 otherwise undetected SARS-CoV-2 infection. Clinical data and test results were analyzed along with community incidence and wastewater viral loads. Correlations were calculated using Spearman test, and clinical implications of false-positive results were assessed.

**Results:**

Among 75 667 screening tests performed on 42 666 patients (21 591 [50.6%] female; median [IQR] age, 64 [45-76] years), 761 patients (1.2% of tests) had positive results. These were classified as true-positive results in 483 patients (63.5%) and false-positive results in 278 patients (36.5%). Among patients with false-positive test results, 139 patients (50.0%) experienced unnecessary isolation, 46 patients (16.5%) were exposed to patients with true-positive SARS-CoV-2 test results by cohorting, and 9 patients (3.2%) received delayed interventions. Screening test positivity correlated with local incidence of SARS-CoV-2 infections and wastewater viral loads. A total of 93.7% of positive test results were recorded during weeks with high community incidence (ie, >150 events per 100 000 inhabitants), in which the proportion of patients with false-positive test results was lower (249 of 709 patients [35.1%]) compared with weeks with low community incidence (ie, <150 events 100 000 inhabitants), with 29 of 52 patients (55.8%) having false-positive results.

**Conclusions and Relevance:**

In this cohort study, universal SARS-CoV-2 screening detected asymptomatic carriers of SARS-CoV-2 infection, aligned with community incidence, and thus may support infection prevention and control measures. However, the unintended clinical outcomes, particularly during times of low incidence, necessitate careful contextual implementation.

## Key Points

**Question:**

What diagnostic value and clinical outcomes are associated with universal SARS-CoV-2 screening of asymptomatic patients, and is test positivity correlated with community incidence levels and wastewater viral loads?

**Findings:**

In this cohort study including 75 667 tests among 42 666 patients, 1.2% had positive results, 36.5% of which were false positives. Test positivity was strongly correlated with community incidence and wastewater viral load, and false-positive results were associated with unnecessary isolation, increased exposure risk, and delays in interventions.

**Meaning:**

Universal screening may support infection control during high community transmission but has limited benefit and potential harms during low-incidence periods.

## Teaser

This retrospective cohort study assesses the diagnostic value and clinical outcomes of universal SARS-CoV-2 screening of asymptomatic patients and the correlation of test positivity with community incidence levels and wastewater viral loads.

## Introduction

Health care environments represent high-risk settings for SARS-CoV-2 transmission, with secondary attack rates in shared hospital rooms comparable to those reported in households.^1^ Transmission rates may differ depending on circulating variants and vaccination rates of patients and health care workers.^2^ Early detection of SARS-CoV-2 infection by universal testing of asymptomatic patients may mitigate nosocomial spread and is supported by the European Society of Clinical Microbiology and Infectious Diseases^3^ when community incidence is high (ie, >300 infections per 100 000 population in 14 days or >150 infections per 100 000 population in 7 days) while considering the balance of benefits and potential harms. Others suggest that systematic admission screening of asymptomatic patients would be useful at a clinically meaningful number-needed-to-test (NNT) threshold of 1:100 or lower,^4^ allowing for more people to be diagnosed with infection at an earlier stage of disease. Drawbacks of systematic testing of asymptomatic patients may include delays in patient placement and transitions of care and capacity strains from prolonged lengths of stay, as well as strains on testing facilities.^5^ False-positive test results, which may increase in low-prevalence settings,^6,7^ can result in an unnecessary delay of investigations and medical procedures, as well as unnecessary admissions to COVID-19 isolation wards with subsequent nosocomial exposure.^8^

As knowledge on the benefits and risks of universal screening for SARS-CoV-2 in asymptomatic patients remains conflicting, we analyzed our data on a systematic screening strategy implemented at our institution over an 89-week period. This study evaluates the diagnostic yield of systematic SARS-CoV-2 screening performed on asymptomatic patients on admission and during their hospital stay in correlation with community 7-day incidence and wastewater viral load and assesses how false-positive results are associated with patient management.

## Methods

### Study Design and Setting

This cohort study was deemed a nonresearch, quality-improvement project not requiring approval from the Ethics Committee Northwest and Central Switzerland, thus not requiring informed consent. The Strengthening the Reporting of Observational Studies in Epidemiology (STROBE) reporting guideline was followed. This retrospective quality-control cohort study was performed from January 2024 to February 2025 at the University Hospital Basel in Switzerland, a tertiary academic care center admitting more than 40 000 adult patients annually.

### Study Population

Starting February 8, 2021, our hospital implemented a universal SARS-CoV-2 screening policy for all asymptomatic patients on admission and at regular intervals during their hospital stay. Patients in quarantine or isolation, patients discharged the same day, and patients with COVID-19 during their hospital stay were excluded from screenings, while all others were screened. For this analysis, we included all patients with systematic screenings for SARS-CoV-2 infection from February 8, 2021, to July 5, 2021, and from August 25, 2021, to December 5, 2022, at the University Hospital Basel. Testing was suspended between July 6, 2021, and August 25, 2021, due to the low incidence in the population.

### Screening for SARS-CoV-2

The screening intervals for universal testing of asymptomatic patients for SARS-CoV-2 were adjusted over time (eFigure 1 in Supplement 1). Patients were screened by collecting saliva samples performed either at the emergency department or on the wards, depending on the reason for admission.

The SARS-CoV-2 Cobas Tests (Roche) was used to analyze saliva samples (turnaround time of approximately 6 hours). Additionally, multiplex polymerase chain reaction (PCR) tests, including SARS-CoV-2 Biofire and the Xpert Xpress-CoV-2/Flu/RSV-Plus system (Cepheid) (turnaround time of approximately 90 minutes), as well as an in-house PCR test were available as confirmation tests in patients with an equivocal first test results (turnaround time of approximately 9 hours).

### Definitions

The results of the SARS-CoV-2 screening tests were categorized as negative if the cycle threshold (Ct) value was at least 40, as positive if the Ct value was less than 30, and as equivocal if the Ct value was between 30 and less than 40. The Ct value cutoff of 30 was chosen based on reports suggesting that a Ct value of approximately 30 is generally considered indicative of low infectivity for SARS-CoV-2 in saliva samples.^9,10^ Per institutional policy, patients with equivocal test results received a nasopharyngeal swab conducted within 72 hours. Based on the second test result, patients were categorized as having true-positive results if the Ct value was less than 30 or a quantitative SARS-CoV-2 test revealed more than 10 000 viral copies/mL and as having false-positive results if the Ct value was 30 or greater, a quantitative test detected fewer than 10 000 viral copies/mL, or the Biofire result was negative. Patients were also classified as having false-positive results if no further test was conducted within 72 hours (eFigure 2 in Supplement 1), with additional sensitivity analyses being performed assigning patients with equivocal test results and without confirmatory testing within 72 hours to the true-positive group for comparisons of characteristics between patients with true- and false-positive test results and correlations between test-positivity and 7-day incidence, as well as wastewater viral loads.

### Institutional Infection Prevention and Control Policy

Our institution implemented several strategies to prevent further in-hospital spread of SARS-CoV-2. These included visitor restrictions and mask requirements (eFigure 1 in Supplement 1). Patients with confirmed SARS-CoV-2 infection were admitted to dedicated cohort wards, allocated to single rooms, or placed in multibedded rooms (beyond the cohort ward) together with other patients with SARS-Co2 infection, where combined contact and enhanced droplet precautions were implemented.

### Data Collection

Medical record review was performed to collect pertinent clinical data to assess potential negative implications for patients with false-positive SARS-CoV-2 test results. These included demographics, data related to the hospital stay (ward type, admission and discharge date, patient contacts, medication, and isolation dates), and screening-related data (screening method, result, and date). The delay of interventions was assessed by performing medical record review. Interventions were categorized as being delayed if indicated by the treating physician in the medical record but not received.

For the regional epidemiology and wastewater viral loads during the study period, publicly available cantonal data were downloaded from Open Data Basel-Stadt,^11^ analyzed, and reported as weekly incidence rates per 100 000 inhabitants (“Coronavirus [Covid-19]: Fallzahlen und Inzidenzen Basel-Stadt”) and weekly median copies per mL (“Coronavirus [Covid-19]: Virenmonitoring im Abwasser”), respectively. Wastewater monitoring for SARS-CoV-2 in the canton of Basel-Stadt started in July 2021.^12^

### Outcomes

The primary end points of this study were the proportions of positive and false-positive SARS-CoV-2 test results, as well as the NNT to find 1 otherwise undetected SARS-CoV-2 infection. Secondary end points of this study were the implications of false-positive test results in terms of exposure to patients with SARS-CoV-2 infection by transfer to the cohort wards of multibedded rooms, administration of treatment for SARS-CoV-2 infection (eg, nirmatrelvir and ritonavir, remdesivir, dexamethasone, budesonide tocilizumab, baricitinib, casirivimab and imdevimab, sotrovimab, or tixagevimab and cilgavimab), and postponement of planned medical procedures (including diagnostics, interventions, surgery, and medical treatment, such as chemotherapy).

### Statistical Analysis

Categorical variables were summarized as counts and proportions, continuous variables as medians and IQRs. The χ^2^ test or Fisher exact (as appropriate) and Mann-Whitney *U* test were applied for comparisons of proportions and continuous variables. These tests were used to compare patient characteristics based on positive and negative results, as well as test positivity between high (ie, >150 infections per 100 000 inhabitants) and low (ie, <150 000 infections per 100 000 inhabitants) community incidence rates. Spearman correlations were calculated to analyze associations between weekly test positivity overall, test positivity of tests performed on admission as well as during follow-up, Ct values, and community 7-day incidence, as well as weekly community wastewater viral load. Sensitivity analyses were performed to assess these associations after exclusion of false-positive test results and to assess associations with false-positive test results. Spearman correlation coefficients were classified as strong at more than 0.60, moderate at 0.40 to 0.59, and weak or negligible at less than 0.40. The NNT was calculated by dividing 1 by the weekly observed test-positivity rate (ie, number of positive test results divided by the total number of tests performed). *P* values were 2-sided, and statistical significance was set at *P* ≤ .05. Statistical analyses were performed using MATLAB version 23.2. (R2023b; MathWorks) and Stata version 16.1 (StataCorp). Charlson Comorbidity Index was calculated using the charlson module.^13^

## Results

During the study period, a total of 78 511 saliva tests were collected from 44 095 admitted patients. Of these, 2568 samples (3.3%) were excluded due to the absence of available Ct values, sampling errors, or invalid dates. Another 276 samples (0.4%) were deemed ineligible due to the patient’s age. The remaining 75 667 PCR saliva tests collected from 42 666 admitted patients (21 591 [50.6%] female; median [IQR] age, 64 [45-76] years) were included in this study (eFigure 3 in Supplement 1).

The number of tests performed within 72 hours after admission was higher (44 818 tests [59.2%]) than the number of follow-up tests performed after 72 hours of hospitalization (30 849 tests [40.8%]). More than 1 test within the first 72 hours after admission was performed either due to a first equivocal test result or due to organizational issues. The overall test positivity rate over the study period was 1.2% (899 tests from 761 patients), with a higher proportion of positive tests (568 tests [63.2%]) resulting from screening performed within the first 72 hours after admission compared with follow-up screening during the hospital stay (331 tests [36.8%]). Testing intervals changed during the study period, A 7-day screening interval was applied during 5 weeks, whereas 5-day (days 0 and 5) and 3- to 5-day (days 0, 3, and 5) screening intervals were used for 47 and 37 weeks, respectively. The proportion of clinically adjudicated true-positive results was similar across these 2 main screening intervals (32.9% and 37.5%, respectively). Most patients entered our institution via the emergency department (19 210 patients [45.0%]), while most tests were performed on medical (28 050 tests [37.1%]) and surgical (28 837 tests [38.1%]) wards.

Among 761 patients (with 899 positive initial test results), we classified 353 patients (46.4%) as having an equivocal first test result (Ct values >30 to <40). Based on the second test result, 278 of these patients (78.8%) were classified as having false-positive results and 75 patients (21.2%) were classified as having true-positive results. Overall, this resulted in 483 patients (63.5%) having true-positive results and 278 patients (36.5%) having false-positive results.

Patients with positive test results for SARS-CoV-2 were older than those with negative results (Table 1). Compared with all patients, the median (IQR) age of patients with false-positive results was generally lower, at 64 (42-76) years, while the median Charlson Comorbidity Index remained consistent across all groups (eTable 1 in Supplement 1). Sensitivity analyses assigning patients with equivocal test results and without confirmatory testing within 72 hours to the group of true-positive results failed to confirm differences between patients with true- and false-positive test results in terms of age (eTable 2 in Supplement 1).

**Table 1.** Baseline Characteristics of Patients Hospitalized and PCR Test Results for SARS-CoV-2 Saliva Tests

Characteristics	No. (%)			*P* value
	Total	Negative results	Positive results	
Tests, No.	75 667	74 768	899	NA
Patients, No.	42 666	41 905	761	NA
Ward				
Medicine	28 050 (37.1)	27 753 (37.1)	297 (33.0)	<.001
Surgery	28 837 (38.1)	28 493 (38.1)	344 (38.3)	
Gynaecology	7718 (10.2)	7637 (10.2)	81 (9.0)	
Emergency department	10 904 (14.4)	10 727 (14.3)	177 (19.7)	
Other or unknown	158 (0.2)	158 (0.2)	0 (0.0)	
Age, median (IQR), y	64 (45-76)	63 (45-76)	66 (48-77)	.008
Sex				
Female	21 591 (50.6)	21 249 (50.7)	342 (44.9)	.002
Male	21 075 (49.4)	20 656 (49.3)	419 (55.1)	
Median Charlson-Comorbidity-Index (IQR)	1 (0-3)	1 (0-3)	1 (0-3)	<.001

Abbreviation: NA, not applicable.

### False-Positive Test Results

Among 278 patients with a false-positive SARS-CoV-2 test results, 139 patients (50.0%) were placed in isolation during their hospital stay. Of these, 46 patients (16.5%) were placed in cohort wards where they had direct contact and thus were exposed to patients with true-positive SARS-CoV-2 infection (Table 2). Elective surgical procedures and interventions were cancelled or postponed for 9 patients (3.2%) (Table 2). According to hospital internal guidelines, antiviral and immunomodulating drugs were administered to 70 patients (25.2%) based on symptoms initially considered compatible with COVID-19, which were later reassessed as being consistent with other underlying conditions.

**Table 2.** Outcomes in Patients With False-Positive SARS-CoV-2 Test Results in Patients

Outcome	Patients, No. (%) (n = 278)
Isolation	139 (50.0)
Unnecessary cohorting with exposure to patients with COVID-19	46 (16.5)
Medication administered ≥1 time during hospital stay^a^	
Dexamethasone	70 (25.2)
Remdesivir	67 (24.1)
Tocilizumab	9 (3.2)
Budesonide	3 (1.1)
Sotrovimab	2 (0.7)
Baricitinib	1 (0.4)
Tixagevimab and cilgavimab	1 (0.4)
Casirivimab und imdevimab	0
Postponement or cancellation of planned interventions	
Any	9 (3.2)
Cancellation of elective surgical procedure	6 (2.2)
Postponement of coronary arteriography	1 (0.4)
Cancellation of continuous positive airway pressure device adjustment	1 (0.4)
Postponement of neurological examination (visual evoked potentials)	1 (0.4)
Restricted therapy	
Any	3 (1.1)
Restricted chemotherapy for mucosa-assisted lymphoid tissue lymphoma^b^	1 (0.4)
Test result supporting indication for cesarean section	1 (0.4)
Restricted patient evaluation after surgery	1 (0.4)

^a^ Some patients received several of the listed drugs.

^b^ The patient’s planned rituximab/bendamustin cycle was administered without bendamustin)

### Correlations of Test-Positivity and Ct Values With Community 7-Day Incidence and Wastewater Viral Loads

**Figure 1.**
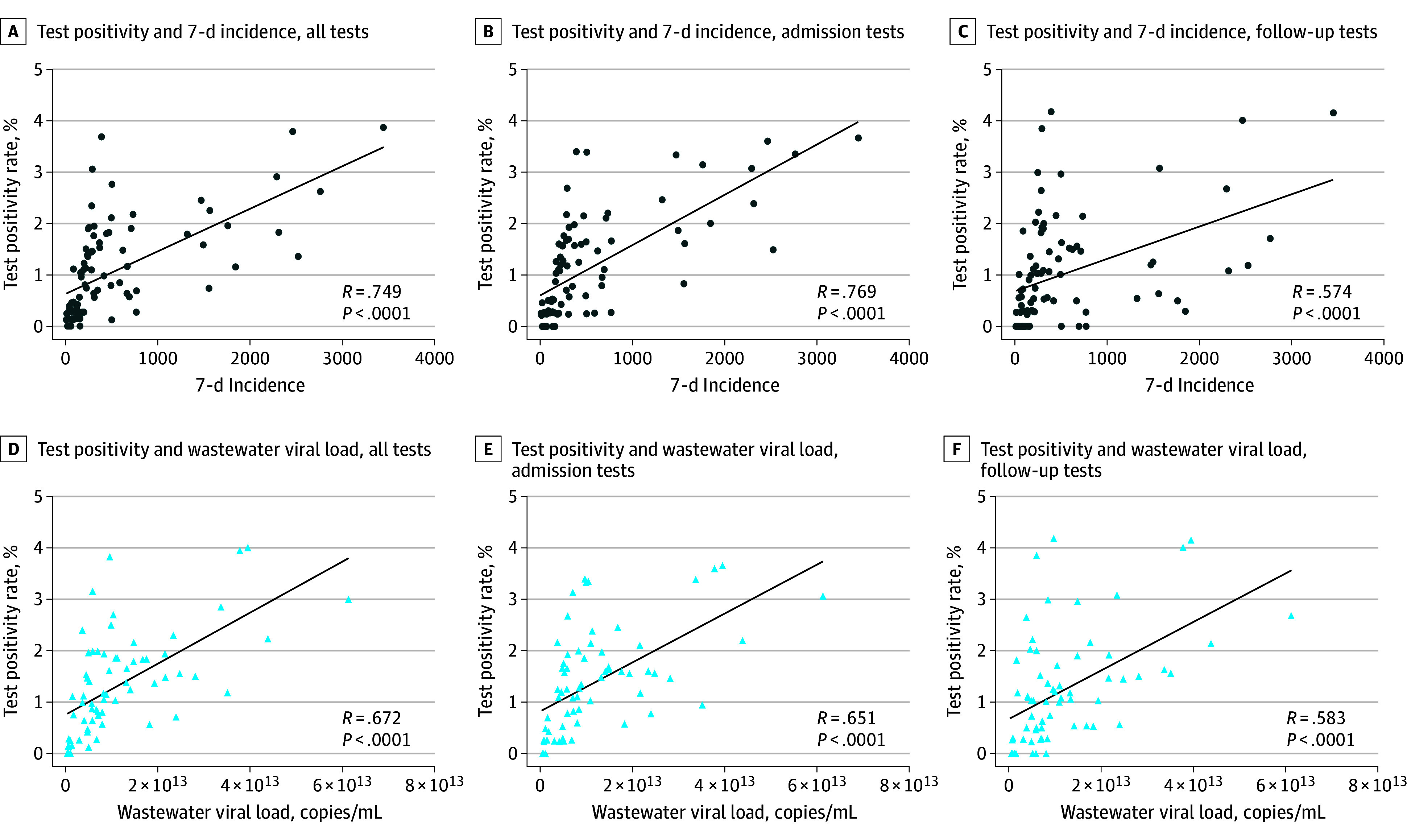
Linear Regression Analysis of Test-Positivity, Wastewater Viral Load, and 7-Day Incidence in Basel-Stadt, Switzerland R indicates Spearman ρ. Incidence is given as infections per 100 000 population.

**Figure 2.**
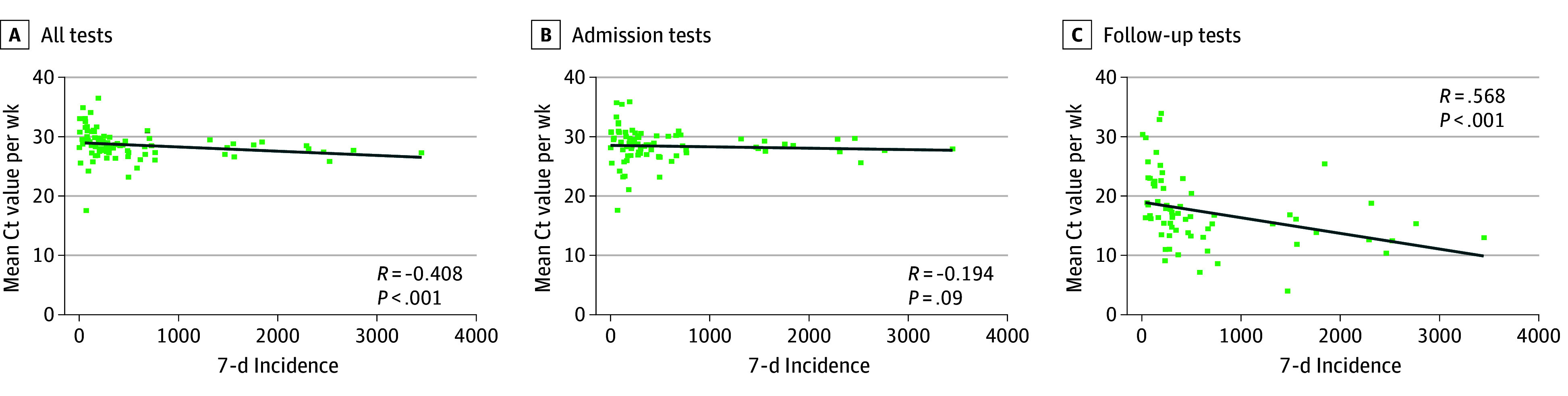
Linear Regression Analysis of Mean Cycle Threshold (Ct) Values of Screening Tests and 7-Day Incidence in Basel-Stadt, Switzerland R indicates Spearman ρ. Incidence is given as infections per 100 000 population.

Test-positivity of systematically screened patients correlated with the local incidence of SARS-CoV-2 infections, as well as the SARS-CoV-2 viral load in the local wastewater (Figure 1, A and D; eFigure 4 in Supplement 1), while local incidence correlated with wastewater viral loads (Spearman correlation, 0.733). Correlations between test-positivity, local 7-day incidence, and wastewater viral load tended to be stronger for admission tests compared with follow-up tests (Figure 1B, C, E, and F). Ct values did not strongly correlate with the community 7-day incidence (Figure 2).

After excluding false-positive test results, we observed stronger correlations of tests performed on admission and during the follow-up period combined, as well as tests performed on admission or during follow-up, with both 7-day incidence and wastewater viral load (eTable 3 in Supplement 1). False-positive test results did not correlate strongly with community 7-day incidence or wastewater viral load (eTable 3 in Supplement 1). Sensitivity analyses assigning patients with equivocal test results and without confirmatory testing within 72 hours to the group of true-positives confirmed these correlations, with the exception of the correlations identified between test-positivity excluding true-positive results of follow-up tests with 7-day incidence and wastewater viral load (eTable 4 in Supplement 1).

### Correlations of NNT With Community 7-Day Incidence and Wastewater Viral Loads

A strong correlation was observed between the NNT and 7-day community incidence (Spearman correlation, −0.706). Wastewater viral loads showed a moderate correlation (Spearman correlation, ¬0.592) (eFigure 5 in Supplement 1).

### Test-Positivity and NNT According to High and Low Community 7-Day Incidence

During the 89-week study period, the community 7-day incidence was defined as high for 60 weeks, in which 55 408 tests (73.2%) were performed, and as low for the remaining 29 weeks, with 20 259 tests (26.8%) performed. During weeks with high community 7-day incidence, a total of 842 positive SARS-CoV-2 test results (93.7% of all positive tests) were recorded (Table 3). After calculating the NNT (eTable 5 in Supplement 1), we found that 760 positive SARS-CoV-2 test results (84.5%) were conducted during the 41 weeks when the NNT was 1:100 or lower, and 139 positive test results (15.5%) were performed during 42 weeks when the NNT was greater than 1:100 (Table 3). During phases of low community 7-day incidence, the proportion of false-positive results among all positive results was higher, with 29 false positives among 52 positive results (55.8%) compared with phases with high 7-day community incidence (249 false-positive results of 709 positive results [35.1%]) (Table 3).

**Table 3.** Screening Test Results According to Community 7-Day Incidence Levels and Number Needed to Screen

Test results	Community 7-d incidence, No. (%)		*P* value	No. needed to screen		*P* value
	>150 per 100 000 (n = 55 408)	<150 per 100 000 (n = 20 259)		<100 (n = 40 679)	>100 (n = 34 988)	
Negative	54 566 (98.5)	20 202 (99.7)	<.001	39 919 (98.1)	34 849 (99.6)	<.001
Positive	842 (1.5)	57 (0.3)		760 (1.9)	139 (0.4)	
True-positive	460 (64.9)	23 (44.2)	.004	NA	NA	NA
False-positive	249 (35.1)	29 (55.8)		NA	NA	

Abbreviation: NA, not applicable.

## Discussion

This cohort study investigated the diagnostic yield and implications of systematic SARS-CoV-2 screening for asymptomatic hospitalized patients during the COVID-19 pandemic. Among more than 75 000 screening tests, 1.2% had positive results, of which 63.5% were classified as true positives. False-positive screening results, accounting for 36.5% of all positive results, were associated with unintended consequences, such as unnecessary isolation, exposure to infected patients through cohorting, unnecessary administration of antivirals and immunomodulating drugs, and delayed medical procedures. Screening outcomes correlated strongly with local incidence and wastewater viral loads.

Our findings align with prior research suggesting that universal SARS-CoV-2 screening in health care settings can help identify asymptomatic carriers, thereby preventing nosocomial transmission during periods of high community 7-day incidence. Our test-positivity rate of 1.2% was relatively low compared with other studies that reported significantly varying results depending on the study design, patient population, regional prevalence of COVID-19, timing of testing (on admission vs during hospital stay), and other nonpharmaceutical interventions already in place.^14,15,16^ Positivity rates in similar studies have shown significant variability, ranging from as low as 0.005%^17^ to as high as 3.2%^18^ and 6.8%^19^ when screening is performed on admission, and from 1.4% to 2.1%^20^ when performed during the hospital stay.

Most positive results were identified on admission (63.2%), emphasizing the importance of early testing to capture the highest-risk period for transmission. However, subsequent follow-up tests conducted after 72 hours still detected 36.8% of all positive test results, indicating decreasing but ongoing risk of infection during hospital stay. This finding is contrasted by the results of another study,^21^ highlighting that testing asymptomatic patients on admission can reduce nosocomial infection rates, whereas additional postadmission testing did not significantly enhance this reduction.

The proportion of false-positive results in our study underscores concerns raised in previous literature regarding the increased likelihood of false-positive test results in low-prevalence settings. False-positive rates of 36.5% in our study align with other reports^6^ documenting the challenges of maintaining high specificity in low-incidence contexts.

Our findings confirm that testing is more efficient and yields more positive results in settings with high community 7-day incidence, which aligns with guidelines of European Society of Clinical Microbiology and Infectious Diseases^3^ and the Society for Healthcare Epidemiology of America.^5^ Our described high correlations between test-positivity rates and wastewater viral loads further support the value of wastewater-based epidemiology as a complementary surveillance tool,^12,22,23,24^ which may be used to guide screening strategies.

Strengths of our study include its comprehensive dataset, with a large number of screening tests performed over an extended time-period, allowing for robust correlation analyses with epidemiological and wastewater data. Analyses of the clinical outcomes associated with false-positive results allow for a more balanced assessment of benefits and risks of such a screening strategy.

### Limitations

This study has some limitations. The retrospective study design and its restriction to a single-center may limit generalizability. Analyses of the unintended outcomes relied on data collected by retrospective medical record review and therefore may not be complete, possibly leading to an underestimation. Furthermore, classification of patients with equivocal test results and missing confirmatory testing within 72 hours may have resulted in misclassification, a limitation addressed by sensitivity analyses accounting for the possibility of these patients having either true- or false-positive test results. Ct values can vary significantly among different PCR assays and target genes, leading to inconsistent results and potential misinterpretation of a patient’s infectious status. Testing intervals changed during the study period, potentially influencing consistency of the data and the interpretation of the results. A 7-day screening interval was applied only briefly (5 weeks), whereas 5-day (days 0 and 5) and 3- to 5-day (days 0, 3, and 5) screening intervals were used for substantially longer periods (47 and 37 weeks, respectively). While increased testing frequency might be expected to increase the proportion of true-positive detections, the proportion of clinically adjudicated true-positive results was similar across the 2 main screening intervals (32.9% vs 37.5%), suggesting no meaningful impact on the primary outcomes. In addition, protocol changes were closely aligned with epidemiologic trends, limiting the added value of stratified analyses. Wastewater viral load data were not available for the full study period, with measurements missing between February 8, 2021, and July 5, 2021. Because wastewater surveillance is independent of diagnostic testing policies and population testing uptake, we nonetheless consider it to provide complementary information to reported community incidence. Accordingly, available wastewater data from August 25, 2021, to December 5, 2022, were used to characterize viral activity in the population during the remainder of the study period. We further acknowledge that our findings may not be generalizable to other settings with different epidemiological contexts in terms of circulating SARS-CoV-2 variants and access to vaccines and that diagnostic yield of screening tests may thus change over the course of the pandemic. Furthermore, this study was not designed to evaluate the associations of screening strategies with nosocomial SARS-CoV-2 transmission, as the study period did not include intervals without screening, preventing comparisons between periods with and without screening implementation.

## Conclusions

The findings of this cohort study suggest that while systematic screening was associated with supporting infection prevention and control efforts during high community incidence, its associated effectiveness diminishes during periods of low incidence and may result in unintended negative consequences for patient treatment. Our findings highlight the considerable unintended outcomes associated with false-positive results, which can strain health care systems and adversely affect patient outcomes. These results emphasize the importance of context-driven implementation, in which screening efforts are aligned with epidemiological trends and resource availability.

## Notes

Supplement 1. eTable 1. Baseline characteristics of cases hospitalized (N=42 666). eTable 2. Baseline characteristics of cases hospitalized (N=42 666) after sensitivity analyses, assigning patients with equivocal test results and without confirmatory testing within 72 hours to the group of true-positives eTable 3. Comparison of Spearman’s correlation coefficients between test-positivity (overall, after exclusion of false positive and after exclusion of true positive cases/tests), 7-day incidence/100 000 inhabitants of canton of Basel-Stadt of SARS-CoV-2 infection and wastewater viral loads eTable 4. Sensitivity analyses of the comparison of Spearman’s correlation coefficients (after assigning patients with equivocal test results and without confirmatory testing within 72 hours to the group of true-positives) between test-positivity (overall, after exclusion of false positive and after exclusion of true positive cases/tests), 7-day incidence/100 000 inhabitants of canton of Basel-Stadt of SARS-CoV-2 infection and wastewater viral loads eTable 5. Performed tests at hospital, test-positivity and calculated NNS eFigure 1. Timeline of asymptomatic SARS-CoV-2 testing policies at University Hospital of Basel, public measures and leading SARS-CoV-2 variant, while the red arrows correspond to the study period eFigure 2. Workflow of the categorization of the results of SARS-CoV-2 screening tests eFigure 3. Fow-chart of study inclusion of tests and cases eFigure 4. Screening test-positivity, local weekly incidence per 100 000 inhabitants and wastewater viral load (copies/ml) over time during the study period eFigure 5. Number needed to screen, local weekly incidence per 100 000 inhabitants and wastewater viral load (copies/mL) over time during the study period

Supplement 2. Data Sharing Statement
