# Supplementary material for: Diagnostic Value and Outcomes of Systematic SARS-CoV-2 Screening in Asymptomatic Patients
Source: JAMA Netw Open. 2026 Apr 8;9(4):e265867. doi: 10.1001/jamanetworkopen.2026.5867 (PMC13063075; doi:10.1001/jamanetworkopen.2026.5867)
Supplement: Supplement 1. — eTable 1. Baseline characteristics of cases hospitalized (N=42 666). eTable 2. Baseline characteristics of cases hospitalized (N=42 666) after sensitivity analyses, assigning patients with equivocal test results and without confirmatory testing within 72 hours to the group of true-positives eTable 3. Comparison of Spearman’s correlation coefficients between test-positivity (overall, after exclusion of false positive and after exclusion of true positive cases/tests), 7-day incidence/100 000 inhabitants of canton of Basel-Stadt of SARS-CoV-2 infection and wastewater viral loads eTable 4. Sensitivity analyses of the comparison of Spearman’s correlation coefficients (after assigning patients with equivocal test results and without confirmatory testing within 72 hours to the group of true-positives) between test-positivity (overall, after exclusion of false positive and after exclusion of true positive cases/tests), 7-day incidence/100 000 inhabitants of canton of Basel-Stadt of SARS-CoV-2 infection and wastewater viral loads eTable 5. Performed tests at hospital, test-positivity and calculated NNS eFigure 1. Timeline of asymptomatic SARS-CoV-2 testing policies at University Hospital of Basel, public measures and leading SARS-CoV-2 variant, while the red arrows correspond to the study period eFigure 2. Workflow of the categorization of the results of SARS-CoV-2 screening tests eFigure 3. Fow-chart of study inclusion of tests and cases eFigure 4. Screening test-positivity, local weekly incidence per 100 000 inhabitants and wastewater viral load (copies/ml) over time during the study period eFigure 5. Number needed to screen, local weekly incidence per 100 000 inhabitants and wastewater viral load (copies/mL) over time during the study period [file jamanetwopen-e265867-s001.pdf]

Supplemental Online Content

Weiss M, Urwyler P, von Rotz M, et al. Diagnostic value and outcomes of systematic SARS-CoV-2 screening in asymptomatic patients. *JAMA Netw Open*. 2026;9(4):e265867. doi:10.1001/jamanetworkopen.2026.5867

eTable 1. Baseline characteristics of cases hospitalized (N=42’666)
eTable 2. Baseline characteristics of cases hospitalized (N=42 666) after sensitivity analyses, assigning patients with equivocal test results and without confirmatory testing within 72 hours to the group of true-positives
eTable 3. Comparison of Spearman’s correlation coefficients between test-positivity (overall, after exclusion of false positive and after exclusion of true positive cases/tests), 7-day incidence/100 000 inhabitants of canton of Basel-Stadt of SARS-CoV-2 infection and wastewater viral loads
eTable 4.S ensitivity analyses of the comparison of Spearman’s correlation coefficients (after assigning patients with equivocal test results and without confirmatory testing within 72 hours to the group of true-positives) between test-positivity (overall, after exclusion of false positive and after exclusion of true positive cases/tests), 7-day incidence/100’000 inhabitants of canton of Basel-Stadt of SARS-CoV-2 infection and wastewater viral loads
eTable 5. Performed tests at hospital, test-positivity and calculated NNS.
eFigure 1. Timeline of asymptomatic SARS-CoV-2 testing policies at University Hospital of Basel, public measures and leading SARS-CoV-2 variant, while the red arrows correspond to the study period.
eFigure 2. Workflow of the categorization of the results of SARS-CoV-2 screening tests.
eFigure 3. Fow-chart of study inclusion of tests and cases.
eFigure 4. Screening test-positivity, local weekly incidence per 100 000 inhabitants and wastewater viral load (copies/ml) over time during the study period.
eFigure 5. Number needed to screen, local weekly incidence per 100 000 inhabitants and wastewater viral load (copies/mL) over time during the study period

This supplemental material has been provided by the authors to give readers additional information about their work.

© 2026 Weiss M et al. *JAMA Network Open*.

Tables

Characteristics	All cases	True-positive	False-positive	p-value
**Median age, y (IQR)**	64 (45–76)	68 (51–78)	64 (42–76)	0.008*
**Sex, No. (%)**				
**Female**	21'591 (50.6)	221 (45.8)	121 (43.5)	0.596**
**Male**	21'075 (49.4)	262 (54.2)	157 (56.5)	
**Median Charlson-Comorbidity-Index (IQR)**	1 (0-3)	1 (0-3)	1 (0-3)	0.078*

Supplementary table 1. Baseline characteristics of cases hospitalized (N=42'666). *Mann-Whitney-U test, **Fisher-exact-Test.

Characteristics	All cases	True-positive	False-positive	p-value
**Median age, y (IQR)**	64 (45–76)	67 (50–78)	65 (46–76)	0.103*
**Sex, No. (%)**				
**Female**	21'591 (50.6)	268 (46.9)	74 (38.9)	0.064**
**Male**	21'075 (49.4)	303 (53.1)	116 (61.1)	
**Median Charlson-Comorbidity-Index (IQR)**	1 (0-3)	1 (0-3)	1 (0-3)	0.912*

Supplementary table 2. Baseline characteristics of cases hospitalized (N=42'666) after sensitivity analyses, assigning patients with equivocal test results and without confirmatory testing within 72 hours to the group of true-positives. *Mann-Whitney-U test, **Fisher-exact-Test.

	**7d-Incidence**		**Median wastewater viralload**	
**Test-positivity overall (true & false positive cases)**	**r**	**p**	**r**	**p**
Admission & follow-up tests combined	0.749	p<0.001	0.672	p<0.001
Admission tests	0.769	p<0.001	0.651	p<0.001
Follow-up tests	0.574	p<0.001	0.583	p<0.001
**Test-positivity excluding false positive cases**				
Admission & follow-up tests combined	0.797	p<0.001	0.705	p<0.001
Admission tests	0.790	p<0.001	0.679	p<0.001
Follow-up tests	0.629	p<0.001	0.581	p<0.001
**Test-positivity excluding true positive cases**				
Admission & follow-up tests combined	0.487	p<0.001	0.474	p<0.001
Admission tests	0.560	p<0.001	0.523	p<0.001
Follow-up tests	0.211	p=0.048	0.282	p=0.020

Supplementary table 3. Comparison of Spearman's correlation coefficients between test-positivity (overall, after exclusion of false positive and after exclusion of true positive cases/tests), 7-day incidence/100'000 inhabitants of canton of Basel-Stadt of SARS-CoV-2 infection and wastewater viral loads

© 2026 Weiss M et al. *JAMA Network Open*.

	7d-Incidence		Median wastewater viralload	
**Test-positivity excluding false positive cases**				
Admission & follow-up tests combined	0.787	p<0.001	0.707	p<0.001
Admission tests	0.788	p<0.001	0.687	p<0.001
Follow-up tests	0.603	p<0.001	0.614	p<0.001
**Test-positivity excluding true positive cases**				
Admission & follow-up tests combined	0.409	p<0.001	0.376	P=0.002
Admission tests	0.456	p<0.001	0.448	p<0.001
Follow-up tests	0.114	p = 0.286	0.130	p = 0.289

Supplementary table 4. Sensitivity analyses of the comparison of Spearman's correlation coefficients (after assigning patients with equivocal test results and without confirmatory testing within 72 hours to the group of true-positives) between test-positivity (overall, after exclusion of false positive and after exclusion of true positive cases/tests), 7-day incidence/100'000 inhabitants of canton of Basel-Stadt of SARS-CoV-2 infection and wastewater viral loads

Year	Calendar week	Positive tests	Total tests	Test positivity	NNS
2021	2021.1				
	2021.2				
	2021.3				
	2021.4				
	2021.5				
	2021.6	0	166	0.00%	
	2021.7	2	594	0.34%	297
	2021.8	3	655	0.46%	218
	2021.9	2	652	0.31%	326
	2021.10	8	719	1.11%	90
	2021.11	1	696	0.14%	696
	2021.12	3	758	0.40%	253
	2021.13	1	703	0.14%	703
	2021.14	2	677	0.30%	339
	2021.15	4	706	0.57%	177
	2021.16	2	735	0.27%	368
	2021.17	2	729	0.27%	365
	2021.18	2	730	0.27%	365
	2021.19	1	760	0.13%	760
	2021.20	2	730	0.27%	365
	2021.21	1	719	0.14%	719
	2021.22	3	762	0.39%	254
	2021.23	2	814	0.25%	407
	2021.24	2	827	0.24%	414
	2021.25	1	790	0.13%	790
	2021.26	1	737	0.14%	737
	2021.27	0	35	0.00%	
	2021.28				
	2021.29				

© 2026 Weiss M et al. *JAMA Network Open.*

	2021.30				
	2021.31				
	2021.32				
	2021.33				
	2021.34	3	403	0.74%	134
	2021.35	0	419	0.00%	
	2021.36	1	669	0.15%	669
	2021.37	2	710	0.28%	355
	2021.38	1	741	0.13%	741
	2021.39	0	681	0.00%	
	2021.40	0	717	0.00%	
	2021.41	0	350	0.00%	
	2021.42	1	773	0.13%	773
	2021.43	2	800	0.25%	400
	2021.44	2	763	0.26%	382
	2021.45	5	781	0.64%	156
	2021.46	1	816	0.12%	816
	2021.47	5	781	0.64%	156
	2021.48	2	729	0.27%	365
	2021.49	4	698	0.57%	175
	2021.50	6	709	0.85%	118
	2021.51	5	631	0.79%	126
	2021.52	4	580	0.69%	145
2022	2022.1	5	676	0.74%	135
	2022.2	8	692	1.16%	87
	2022.3	11	808	1.36%	73
	2022.4	21	799	2.63%	38
	2022.5	16	873	1.83%	55
	2022.6	16	817	1.96%	51
	2022.7	14	883	1.59%	63
	2022.8	20	814	2.46%	41
	2022.9	20	887	2.25%	44
	2022.10	31	816	3.80%	26
	2022.11	30	774	3.88%	26
	2022.12	31	1063	2.92%	34
	2022.13	19	1060	1.79%	56
	2022.14	21	1101	1.91%	52
	2022.15	18	985	1.83%	55
	2022.16	16	983	1.63%	61
	2022.17	15	1084	1.38%	72
	2022.18	13	1145	1.14%	88
	2022.19	11	1146	0.96%	104
	2022.20	5	1188	0.42%	238
	2022.21	5	1069	0.47%	214
	2022.22	11	1054	1.04%	96
	2022.23	14	1028	1.36%	73

© 2026 Weiss M et al. *JAMA Network Open*.

	2022.24	7	996	0.70%	142
	2022.25	29	1047	2.77%	36
	2022.26	13	1118	1.16%	86
	2022.27	23	1054	2.18%	46
	2022.28	16	1081	1.48%	68
	2022.29	22	1041	2.11%	47
	2022.30	35	948	3.69%	27
	2022.31	14	958	1.46%	68
	2022.32	20	1024	1.95%	51
	2022.33	12	1094	1.10%	91
	2022.34	16	1063	1.51%	66
	2022.35	12	1083	1.11%	90
	2022.36	8	994	0.80%	124
	2022.37	11	1079	1.02%	98
	2022.38	13	1059	1.23%	81
	2022.39	6	1070	0.56%	178
	2022.40	15	980	1.53%	65
	2022.41	20	1106	1.81%	55
	2022.42	11	1123	0.98%	102
	2022.43	16	1104	1.45%	69
	2022.44	24	1022	2.35%	43
	2022.45	34	1109	3.07%	33
	2022.46	19	1076	1.77%	57
	2022.47	22	1143	1.92%	52
	2022.48	21	1105	1.90%	53

Supplementary table 5. Performed tests at hospital, test-positivity and calculated NNS.

© 2026 Weiss M et al. *JAMA Network Open*.

Figures

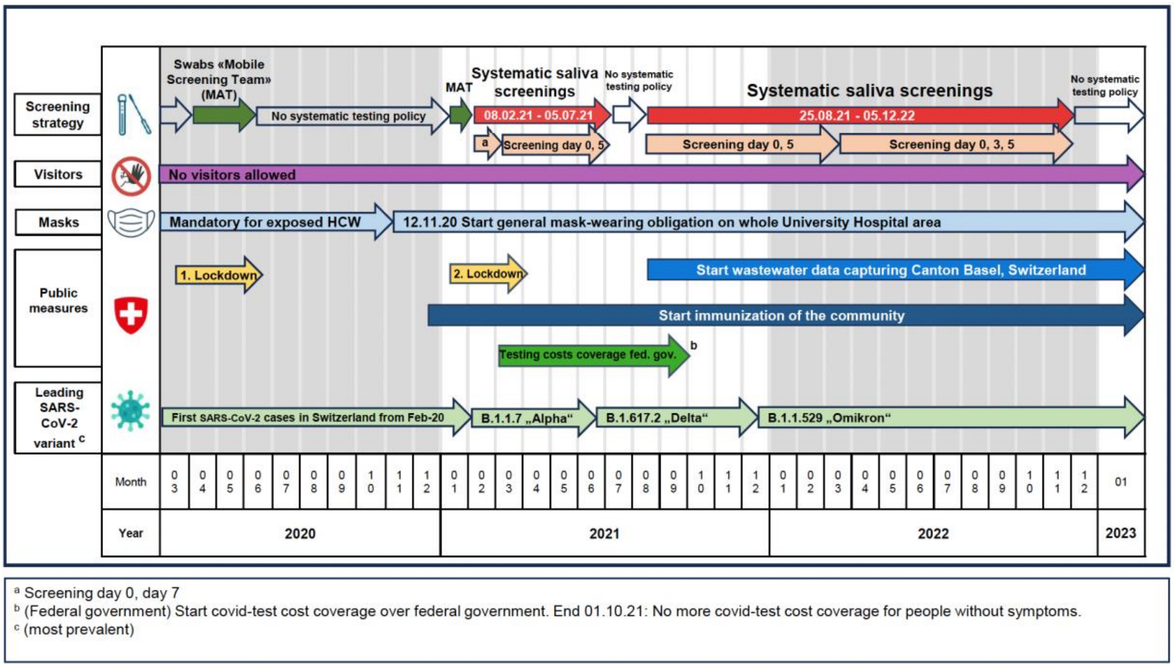

Supplementary figure 1. Timeline of asymptomatic SARS-CoV-2 testing policies at University Hospital of Basel, public measures and leading SARS-CoV-2 variant, while the red arrows correspond to the study period. *Screening day 0, day 7, **Time period of coverage for SARS-CoV-2 tests by federal government, ***(most prevalent).

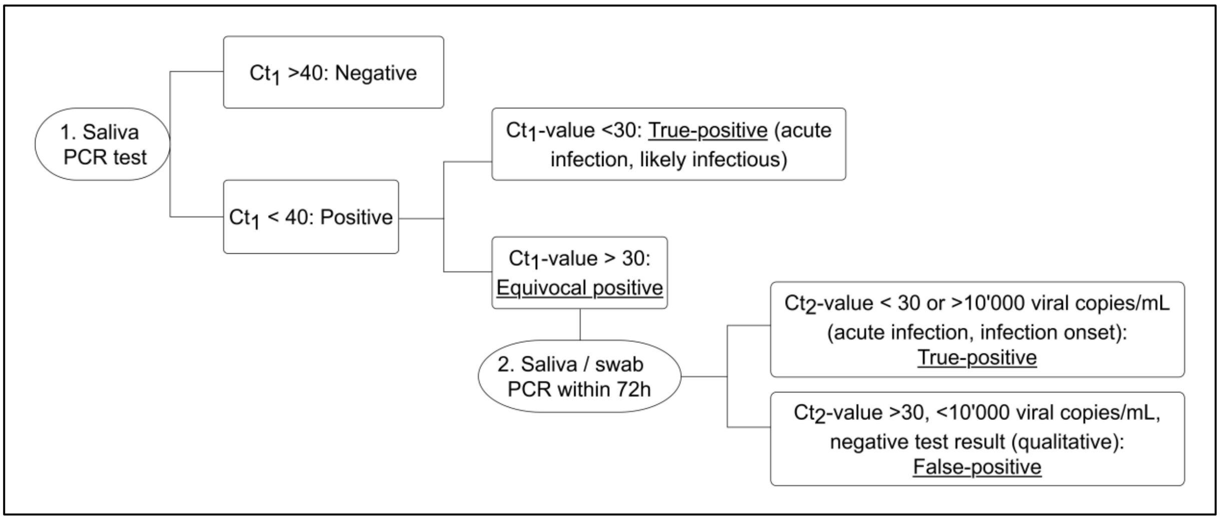

Supplementary figure 2. Workflow of the categorization of the results of SARS-CoV-2 screening tests.

© 2026 Weiss M et al. *JAMA Network Open*.

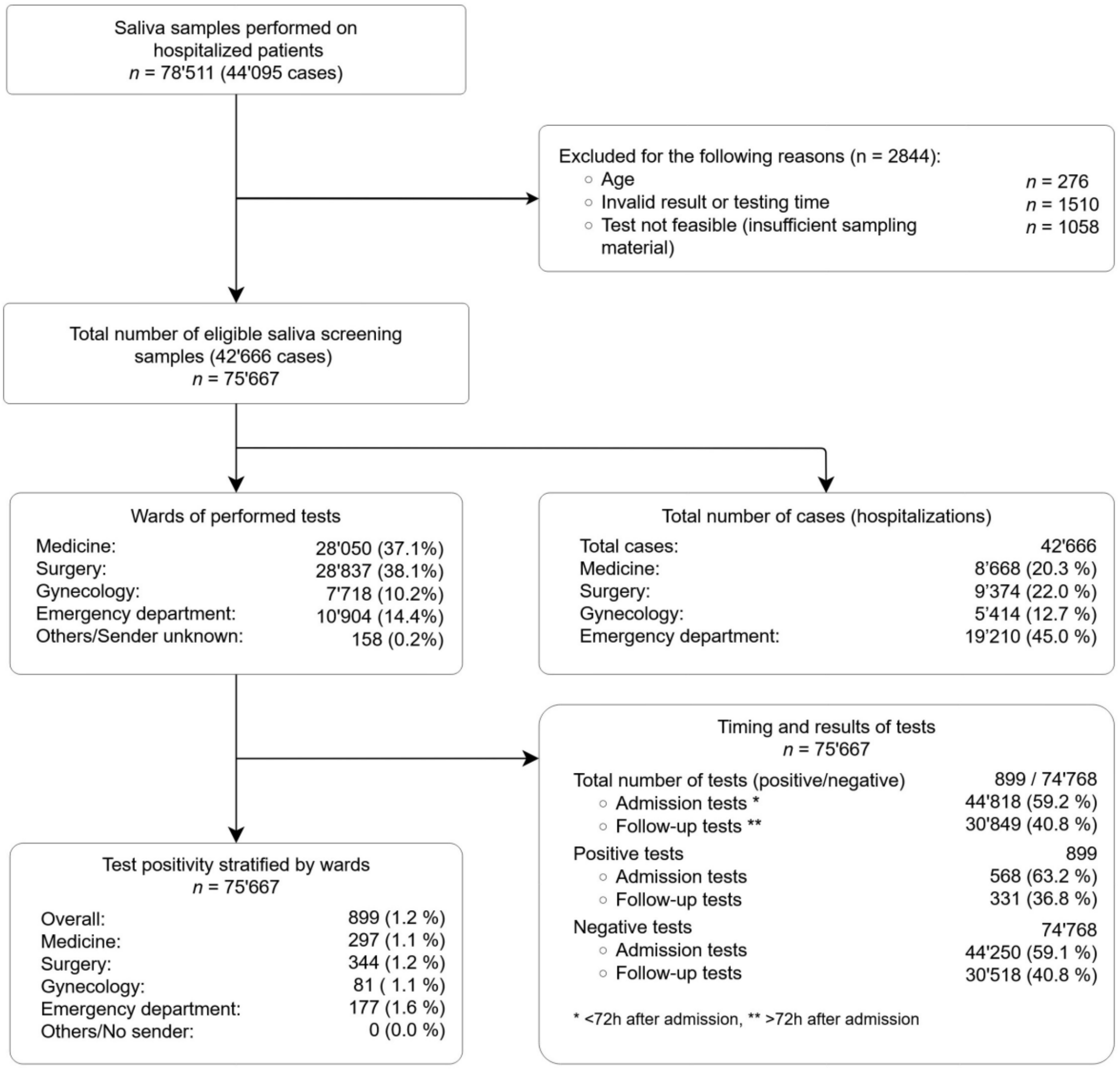

Supplementary figure 3. Fow-chart of study inclusion of tests and cases.

© 2026 Weiss M et al. *JAMA Network Open*.

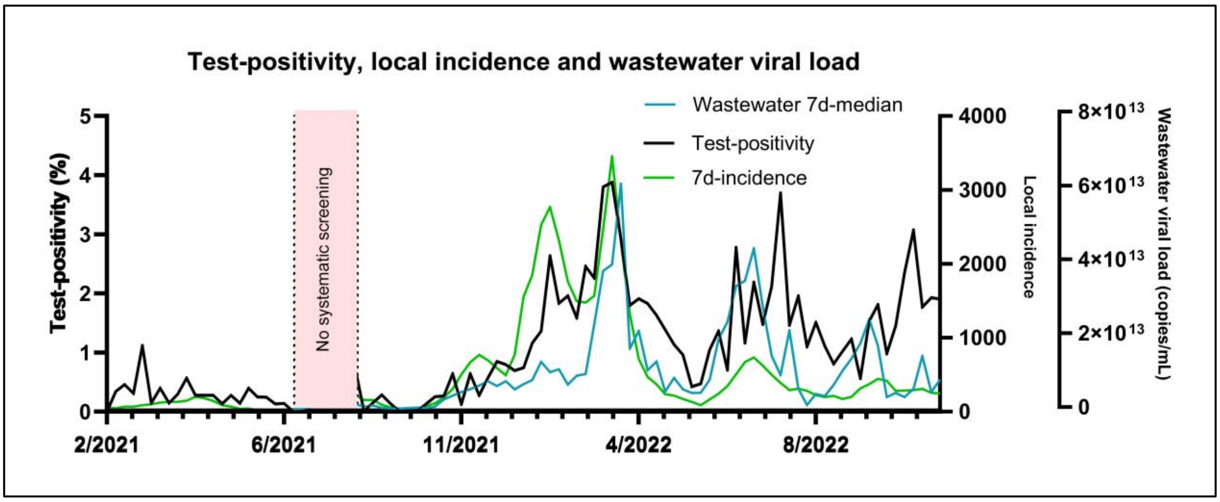

Supplementary figure 4. Screening test-positivity, local weekly incidence per 100’000 inhabitants and wastewater viral load (copies/ml) over time during the study period.

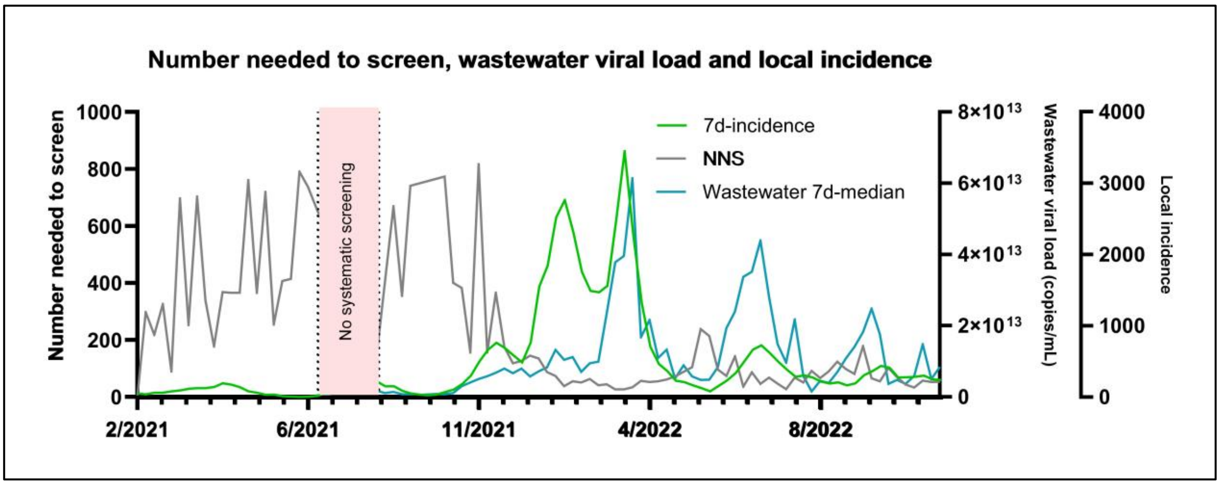

Supplementary figure 5: Number needed to screen, local weekly incidence per 100’000 inhabitants and wastewater viral load (copies/ml) over time during the study period

© 2026 Weiss M et al. *JAMA Network Open*.
