# Supplementary material for: Diagnostic Value and Outcomes of Systematic SARS-CoV-2 Screening in Asymptomatic Patients
Source: JAMA Netw Open. 2026 Apr 8;9(4):e265867. doi: 10.1001/jamanetworkopen.2026.5867 (PMC13063075; doi:10.1001/jamanetworkopen.2026.5867)
Supplement: Supplement 2. — Data Sharing Statement [file jamanetwopen-e265867-s002.pdf]

Data Sharing Statement

Weiss. Diagnostic Value and Outcomes of Systematic SARS-CoV-2 Screening in Asymptomatic Patients. *JAMA Netw Open*. Published April 08, 2026. doi:10.1001/jamanetworkopen.2026.5867

Data
Data available: Yes
Data types: Deidentified participant data, Data (not involving human participants)
How to access data: The data will be provided by the corresponding author upon reasonable request.
When available: With publication

Supporting Documents
Document types: None

Additional Information
Who can access the data: The data will be provided by the corresponding author upon reasonable request.
Types of analyses: The data will be provided by the corresponding author upon reasonable request.
Mechanisms of data availability: The data will be provided by the corresponding author upon reasonable request.
